# Body weight loss after surgery affects the continuity of adjuvant chemotherapy for pancreatic cancer

**DOI:** 10.1186/s12885-019-5621-5

**Published:** 2019-05-02

**Authors:** Yoshifumi Morita, Takanori Sakaguchi, Ryo Kitajima, Satoru Furuhashi, Ryota Kiuchi, Makoto Takeda, Takanori Hiraide, Yasushi Shibasaki, Hirotoshi Kikuchi, Hiroyuki Konno, Hiroya Takeuchi

**Affiliations:** 1 Second Department of Surgery, Hamamatsu University School of Medicine, 1-20-1 Handayama, Higashi-ku, Hamamatsu, 431-3192 Japan; 2 Shizuoka city Shimizu hospital, Shizuoka, Japan; 3 Hamamatsu University School of Medicine, Hamamatsu, Japan

**Keywords:** Adjuvant chemotherapy, Body weight loss, Continuity, Pancreatic cancer

## Abstract

**Background:**

Postoperative chemotherapy is beneficial for many pancreatic cancer patients. However, some patients require dose reduction or the discontinuation of adjuvant chemotherapy because of adverse treatment-related effects. In this study, we aimed to evaluate two main outcomes. First, we evaluated the clinicopathological factors affecting patient disease-free survival (DFS) and overall survival (OS) following upfront surgery. Second, we evaluated the factors that influence the continuity of adjuvant chemotherapy.

**Methods:**

Fifty-four patients with resected pancreatic cancer were enrolled. First, we evaluated the clinicopathological factors affecting postoperative survival using the Kaplan-Meier method and Cox regression method. Next, factors affecting the continuity of adjuvant chemotherapy were analyzed using multiple logistic regression analysis.

**Results:**

Univariate and multivariate analyses revealed that positive LN metastasis (HR (95% CI) 6.329 (2.381–16.95); *p* < 0.001) and relative dose intensity (RDI) < 80% for adjuvant chemotherapy (HR (95% CI) 5.154 (1.761–15.15); *p* = 0.003) were independent predictive factors for DFS. Regarding OS, extended dissection of the nerve plexus around the superior mesenteric artery (SMA) (HR (95% CI) 4.504 (1.721–11.76); *p* = 0.002), positive microscopic surgical margin (HR (95% CI) 5.565 (1.724–17.96); *p* = 0.004), and adjuvant chemotherapy of RDI < 80% (HR (95% CI) 3.534 (1.135–2.667); *p* = 0.029) were also independent predictive factors. Moreover, the level of RDI significantly correlated with DFS and OS. Multiple logistic regression analysis revealed that low RDI was significantly associated with postoperative body weight loss (BWL) ≥ 10%.

**Conclusions:**

The following factors were significantly associated with poor survival: extended dissection of the nerve plexus around the SMA, lymph node metastasis, residual tumor, and RDI of the adjuvant chemotherapy. Patient’s prognosis with adjuvant chemotherapy of RDI < 80% was worse. BWL ≥10% was the most important factor affecting the continuity of adjuvant chemotherapy. Perioperative nutritional intervention is necessary for patients who receive adjuvant chemotherapy for advanced pancreatic cancer.

## Background

Pancreatic cancer is the seventh leading cause of cancer death with more than 450,000 newly diagnosed per year worldwide with increasing rates seen in Europe, North America and Eastern Asian countries [1]. Recent data showed that over the past 10 years, the number of pancreatic cancer patients have been rising at an average annual rate of 0.5% [2]. In the United States, pancreatic cancer is the fourth-leading cause of cancer death with an estimated 55,440 new cases and 44,330 deaths in 2018 [3]. The 5-year survival for pancreatic cancer is the lowest among various cancers, despite recent advances in understanding its biology and improvements in imaging [4]. At present, surgical resection is the only potentially curative approach for pancreatic cancer when the disease is localized. Upfront surgery followed by adjuvant chemotherapy is recommended in patients with potentially resectable pancreatic cancer by the European Society for Medical Oncology (ESMO) and National Comprehensive Cancer Network (NCCN) guidelines [5, 6]. However, even after curative resection, most patients experience recurrence within 2 years and 2/3 patients die within 3 years [7]. Meanwhile, patients with borderline resectable cancer should be considered for neoadjuvant chemotherapy. Although the best regimens in the neoadjuvant setting are still undetermined, FOLFIRINOX or *Nab*-paclitaxel are promising in patients with borderline resectable or locally advanced disease [8, 9].

Adjuvant treatment with gemcitabine had been a gold-standard chemotherapeutic agent for pancreatic cancer [10, 11]. The Japan Adjuvant Study Group of Pancreatic Cancer (JASPAC-01) phase III trial demonstrated that S-1 was more effective for pancreatic cancer than gemcitabine [12]. Recently, the European Study Group for Pancreatic Cancer (ESPAC-4) reported that the combination of gemcitabine and capecitabine was superior to gemcitabine monotherapy in patients with resected pancreatic cancer [13].

Continuity of treatment and maintenance of the dose intensity are important for maximizing the efficacy of adjuvant chemotherapy. However, because of various side effects, reduction in the dose intensity or discontinuation of chemotherapy is sometimes required. Therefore, predicting the therapeutic course of such patients represents a major challenge to providing appropriate disease management. In this retrospective analysis of patients that underwent upfront surgery for pancreatic cancer, we aimed to evaluate two main outcomes. First, we evaluated the clinicopathological factors affecting patient disease-free survival (DFS) and overall survival (OS) following pancreatic resection. Second, we evaluated the factors that influence the continuity of post-surgical adjuvant chemotherapy.

## Methods

### Patients

**Fig. 1:**
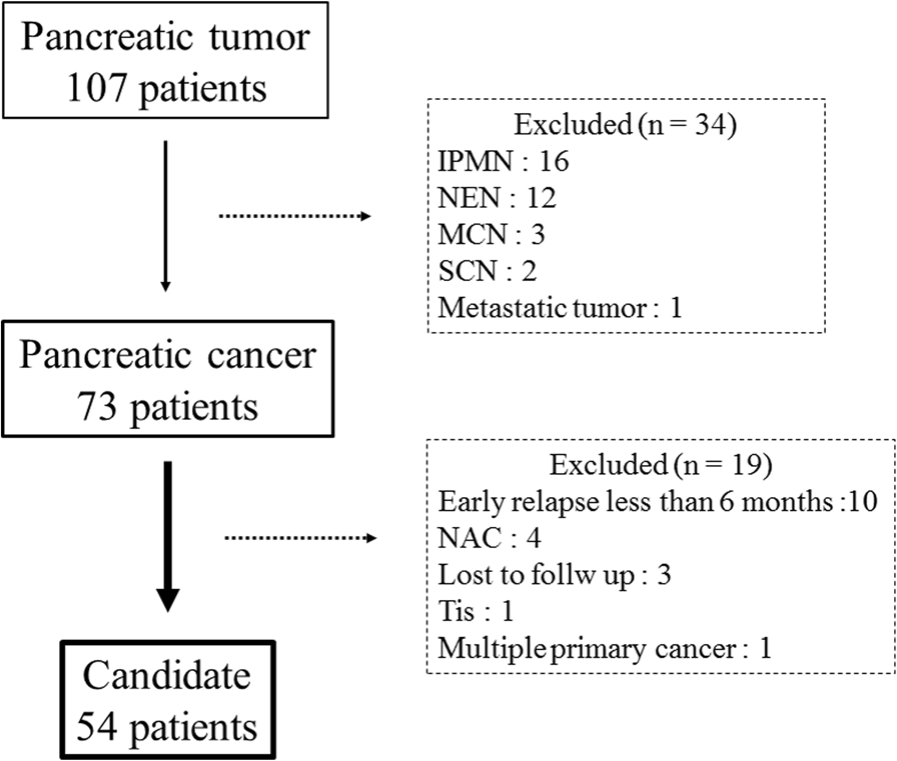
Study population of this retrospective analysis. IPMN: Intraductal papillary mucinous neoplasm, NET NEN: Neuroendocrine tumor neoplasm, MCN: Mucinous cystic neoplasm, SCN: Serous cystic neoplasm, NAC: Neoadjuvant chemotherapy

From 2006 to 2016, 107 patients with pancreatic tumors underwent surgical resection at the Hamamatsu University School of Medicine. Seventy-three of 107 patients were diagnosed as having pancreatic cancer. Of these 73 patients, 19 were excluded from the analysis for the following reasons: 10 had an early relapse of less than 6 months, 4 received neoadjuvant chemotherapy, 3 were lost to follow up, 1 patient had stage 0 disease, and 1 patient had multiple primary malignancies. In total, 54 patients were enrolled in this study (Fig. 1).

TNM stage was assessed according to the 7th edition TNM staging guidelines, published by the American Joint Committee on Cancer [14]. The study protocol conformed to the ethical guidelines of the 1975 Declaration of Helsinki and was approval by the ethical committee of our institution (approval number: 15–318). Written consents to participate in this study were substituted for providing a means to opt out in the website (https://www.hama-med.ac.jp/research/clinical-res/erc/disclosure-info/h29.html) according to the ethics guidelines for clinical studies of the Japanese Ministry of Health, Labour and Welfare (MHLW).

### Outcome

We evaluated two main outcomes. Our primary outcome evaluated the clinicopathological factors affecting patient DFS and OS. DFS was calculated as the duration between the operative day and recurrence. Tumor recurrence was determined by CT, cytology or biopsy. OS was calculated as the duration between the operative day and cancer-related death. The secondary outcome examined the influential factors affecting the continuity of adjuvant chemotherapy including age, operative methods, postoperative complications, blood test results, and body weight loss (BWL). Statistical methods are described in the following section.

### Surgical procedures

Lymphadenectomy for pancreatic head cancer included the anterior and posterior pancreaticoduodenal, pyloric region, hepatoduodenal ligament, common hepatic artery, and superior and inferior pancreatic head lymph nodes. Lymphadenectomy for pancreatic body and tail cancer included celiac trunk, splenic artery, common hepatic artery, splenic hilum, and superior and inferior pancreatic body lymph nodes. Dissection of the nerve plexus around the superior mesenteric artery (SMA) was performed according to the location and extent of the tumor. Concomitant superior mesenteric vein (SMV) and portal vein resection was performed when the involvement of the SMV and portal vein could be safely reconstructed by direct suture or venous graft.

### Postoperative chemotherapy

For the postoperative adjuvant chemotherapy regimen, patients received either gemcitabine or tegafur/gimeracil/oteracil (S-1). For 5 patients, the treatment was changed from either gemcitabine to S-1 or S-1 to gemcitabine. One treatment cycle consisted of weekly intravenous infusions of 1000 mg/ m^2^ of gemcitabine for 1 h, for 3 weeks, followed by a 1-week break. S-1 was administered orally at a dose of 80–120 mg per body surface area (BSA) per day (BSA < 1.25 m^2^, 80 mg/day; 1.25 < BSA < 1.5 m^2^, 100 mg/day; 1.5 m^2^ > BSA, 120 mg/day). Each chemotherapy cycle consisted of S-1 administration for 28 days, followed by 14 days without treatment. For both treatment regimens, the planned treatment period was 6 cycles. Relative dose intensity (RDI) was estimated to be 100% for gemcitabine when the total dose of 18,000 mg/ m^2^ was reached. For S-1, RDI was estimated as 100% when the total S-1 dose of 13,440–20,160 mg, according to BSA (BSA < 1.25 m^2^, 13,440 mg; 1.25 < BSA < 1.5 m^2^, 16,800 mg; 1.5 m^2^ > BSA, 20160 mg), was achieved. When adverse reactions occurred, either the dose was reduced, the dosing interval was adjusted, or administration was temporarily discontinued. Treatment was discontinued or switched to another regimen when the patient showed disease recurrence or adverse events that were uncontrollable by dose modification or temporary cessation of treatment. Adverse events were recorded according to the Common Terminology Criteria for Adverse Events verssion 4.0 [15].

### Statistical analysis

Body weight loss (BWL) was defined as follows:

Percent BWL = (preoperative body weight - body weight at the time of discharge) × 100 / preoperative body weight. Preoperative body weight was measured 2–3 days before surgery. Renal function was measured in terms of creatinine clearance (CCr), calculated according to the formula proposed by Cockroft and Gault [16]. Variables were compared using the χ^2^ test, Fisher’s exact test, or Mann–Whitney’s U test, as appropriate. The cumulative DFS and OS rates were estimated using the Kaplan-Meier method and compared according to the log-rank test. Predictors of outcome were assessed by univariate and multivariate analysis using Cox proportional hazard regression modeling. The stepwise variable selection process was used in the multivariate analysis to identify the most concise model for predicting cumulative survival. Spearman’s rank-correlation coefficient was performed to identify correlation between RDI of adjuvant chemotherapy and DFS and OS. Multivariate logistic regression analysis was performed to identify factors that affect the continuity of adjuvant chemotherapy. A *P* value < 0.05 was defined as statistically significant. SPSS version 24 (IBM Corp., Armonk, NY, USA) was used for all statistical analyses.

## Results

### Clinicopathological characteristics

Clinicopathological characteristics of the entire cohort, as stratified by treatment group, are presented in Table 1. The mean age at diagnosis was 70.4 years (range 45–85 years) with the cohort consisting of 26 males and 28 females. Thirty-nine patients (67.2%) received adjuvant chemotherapy. Eleven patients tolerated an RDI of ≥80%, although the mean RDIs of S-1 and gemcitabine in the adjuvant chemotherapy group were 71.4 and 70.5%, respectively. Fifteen patients (27.8%) did not receive adjuvant therapy after surgery, due to their overall general condition, the presence of comorbidities, or at their own discretion. The RDI for these 15 patients was set to 0. The following clinicopathological characteristics were similar between the 2 groups: surgical method, comorbidities, preoperative BMI, duration of surgery, intraoperative bleeding, number of resected lymph nodes, TNM stage, residual tumor, postoperative diarrhea, clinically-relevant pancreatic fistula and delayed gastric emptying.

**Table 1:** Patient clinicopathological characteristics with or without Adjuvant chemotherapy

	Adjuvant (+) *N* = 39	Adjuvant (−) *N* = 15	*p*
Age (mean ± SD)	69.9 ± 7.8	71.5 ± 7.7	0.499
Sex (Male: Female)	17: 22	9: 6	0.366
Operative method (PD: DP)	28: 11	12: 3	0.733
Comorbidity (Y: N)	27: 12	12: 3	0.515
Preoperative BMI (Kg/m^2^) (mean ± SD)	22.3 ± 2.9	22.4 ± 3.4	0.863
Operation time (min) (mean ± SD)	396 ± 100	394 ± 70	0.955
Intraoperative bleeding (ml) (mean ± SD)	878 ± 626	1102 ± 1339	0.404
Extended dissection of nerve plexus around SMA (Y: N)^a^	12: 27	3: 12	0.515
Concomitant venous reconstruction (Y: N)	5: 34	1: 14	0.461
Number of resected lymph nodes	30 ± 11	28 ± 20	0.730
AJCC Stage (IA: IB: IIA: IIB: III: IV)	2:0:10:23:0:4	3:2:3:7:0:0	0.124
Residual tumor (R0: R1)	32: 7	15: 0	0.171
Postoperative diarrhea (Y: N)^b^	13: 26	7: 8	0.363
Postoperative pancreatic fistula grade B or C	11: 28	1: 14	0.145
Postoperative delayed gastric emptying (Y: N)	7: 32	2: 13	0.697

^a^Extended dissection of nerve plexus around SMA was defined as more than a half

^b^Postoperative diarrhea was defined as increased frequency of defecation one or more times per day

### Disease-free survival and overall survival

**Fig. 2:**
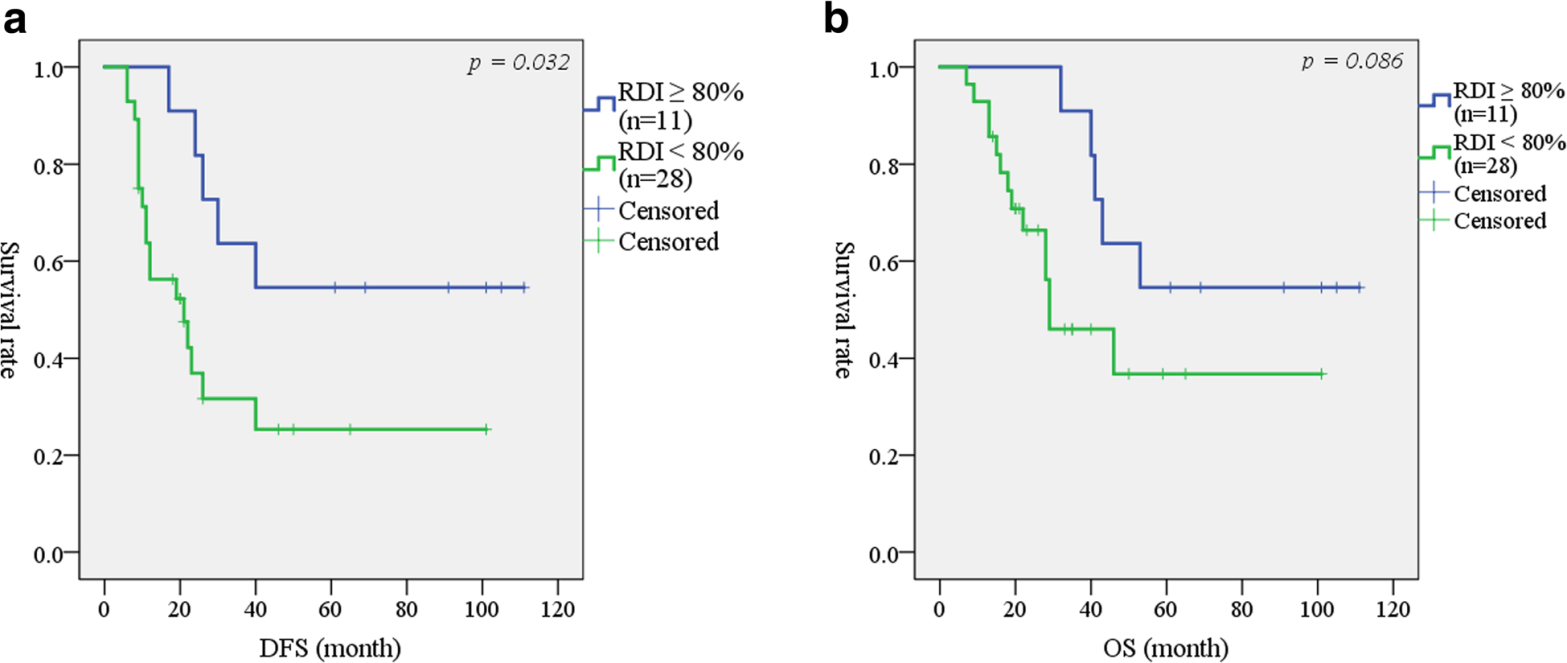
**a** Disease-free survival analysis for the patients who received high (≥ 80%) and low (< 80%) dose intensity of adjuvant chemotherapy. **b** Overall survival analysis for the patients who received high (≥ 80%) and low (< 80%) dose intensity of adjuvant chemotherapy. DFS: Disease-free survival, RDI: Relative dose intensity, OS: Overall survival

The median follow-up time of all patients was 30 months (range 5–111 months). At the time of analysis, 24 (44.4%) of the study patients had died. The median DFS for the entire cohort was 30 months. In the univariate analysis, variables such as extended dissection of the nerve plexus around the SMA (17 months versus 52 months; *p* = 0.007), positive lymph node (LN) metastasis (21 months versus not reached; *p* = 0.005), AJCC Stage (20 months versus not reached; *p* = 0.010), and reduced RDI of the adjuvant chemotherapy (< 80%) (23 months versus not reached; *p* = 0.036) were significantly associated with poor DFS (Table 2). In the multivariate analysis, positive LN metastasis (HR (95% CI) 6.329 (2.381–16.95); *p* < 0.001), and an RDI < 80% for the adjuvant chemotherapy (HR (95% CI) 5.154 (1.761–15.15); *p* = 0.003) were independent predictive factors for DFS (Table 2). This analysis included both adjuvant (+) and adjuvant (−) patients; therefore, for the adjuvant (+) patients, DFS was compared between patients with an RDI ≥80% and those with an RDI < 80%. As shown in Fig. 2a, patients receiving a higher RDI showed significantly better DFS (*p* = 0.032).

**Table 2:** Univariate and multivariate analyses for disease free survival

Variable	Univariate			Multivariate	
	Median				
	No.	survival	*p*-Value	HR (95% CI)	*p*-Value
BWL^a^					
< 10%	28	N.R.			
≥ 10%	26	26	0.218		N.S.
Postoperative diarrhea					
No	34	40			
Yes	20	21	0.194		N.S.
Extended dissection of nerve plexus around SMA^b^					
No	39	52			
Yes	15	17	0.007		N.S.
Residual tumor					
R0	47	40			
R1	7	12	0.144		N.S.
Lymph node metastasis					
Negative	20	N.R.			
Positive	34	21	0.005	3.636 (1.266–9.524)	**0.009**
AJCC Stage					
Stage IA, IB	7	N.R.			
Stage IIA or higher	47	20	0.010		N.S.
RDI^a^ in adjuvant chemotherapy					
≥ 80%	11	N.R.			
< 80%	43	23	0.036	5.421 (1.852–15.86)	**0.002**

^a^BWL (body weight loss): % body weight loss = (preoperative body weight – body weight at the time of discharge) × 100 / preoperative body weight

^b^Extended dissection of nerve plexus around SMA was defined as more than a half

*RDI* Relative dose intensity, *N.R.* not reached

Boldface indicates the statistical significance

**Fig. 3:**
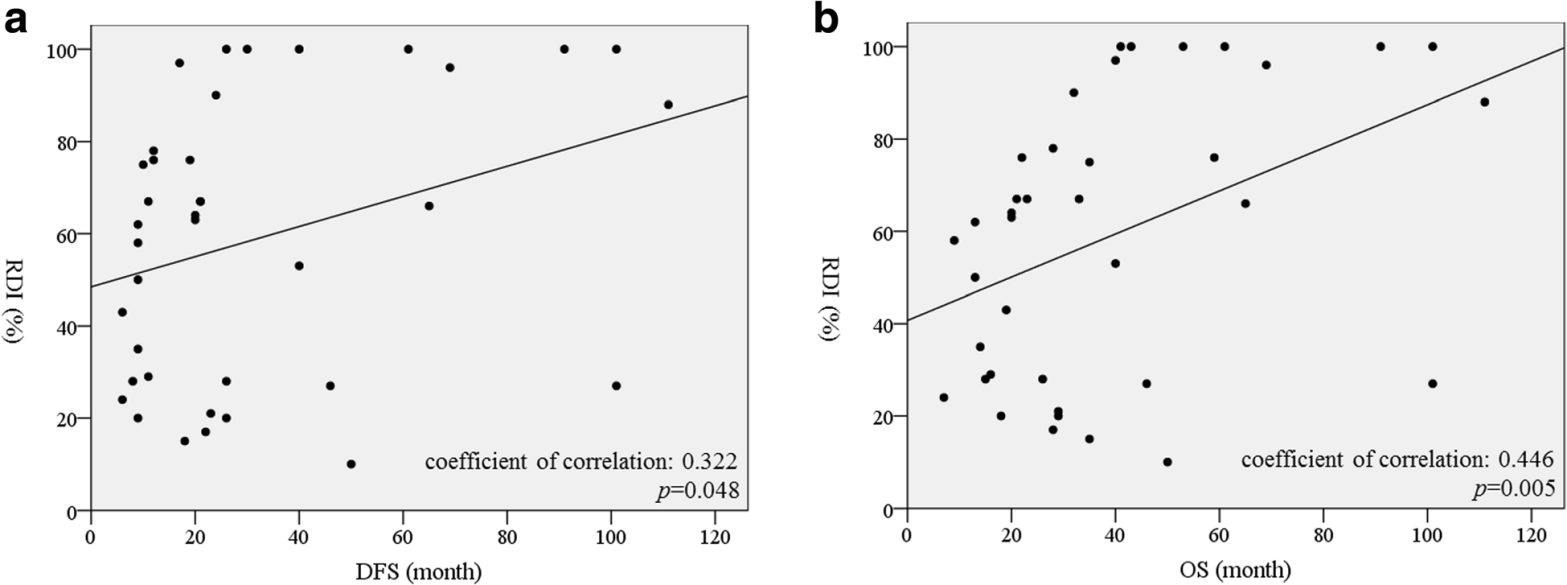
**a** Correlation between the level of relative dose intensity and disease-free survivals. **b** Correlation between the level of relative dose intensity and overall survivals. Black circle indicates each patient with adjuvant chemotherapy. DFS: Disease-free survival, RDI: Relative dose intensity, OS: Overall survival

Regarding OS, variables such as extended dissection of the nerve plexus around the SMA (22 months versus 67 months; *p* = 0.011), AJCC Stage (28 months versus not reached; *p* = 0.026), and reduced RDI of the adjuvant chemotherapy (46 months versus not reached; *p* = 0.045) were significantly worse in the univariate analysis (Table 3). In the multivariate analysis, extended dissection of the nerve plexus around the SMA (HR (95% CI) 4.504 (1.721–11.76); *p* = 0.002), positive microscopic surgical margin (R1) (HR (95% CI) 5.565 (1.724–17.96); *p* = 0.004), and adjuvant chemotherapy of RDI < 80% (HR (95% CI) 3.534 (1.135–2.667); *p* = 0.029) were also independent predictive factors for poor prognosis (Table 3). Similar to trend for DFS, patients who received RDI ≥80% tended to have longer OS than those with an RDI < 80% (*p* = 0.086) (Fig. 2b). Moreover, the level of RDI significantly correlated with DFS and OS for the adjuvant (+) patients, respectively (Fig. 3).

**Table 3:** Univariate and multivariate analyses for overall survival

		Univariate		Multivariate	
		Median			
Variable	No.	survival	*p*-Value	HR (95% CI)	*p*-Value
BWL^a^					
< 10%	28	N.R.			
≥ 10%	26	46	0.163		N.S.
Postoperative diarrhea					
No	34	40			
Yes	20	21	0.379		N.S.
Extended dissection of nerve plexus around SMA^b^					
No	39	67			
Yes	15	22	0.011	3.289 (1.269–8.547)	**0.014**
Residual tumor					
R0	47	53			
R1	7	28	0.095	4.236 (1.336–13.42)	**0.014**
Lymph node metastasis					
Negative	20	67			
Positive	34	40	0.051		N.S.
AJCC Stage					
Stage IA, IB		7	N.R.		
Stage IIA or higher	47	28	0.026		N.S.
RDI in adjuvant chemotherapy					
≥ 80%	11	N.R.			
< 80%	43	46	0.045	4.437 (1.437–13.70)	**0.010**

^a^BWL (body weight loss): % body weight loss = (preoperative body weight – body weight at the time of discharge) × 100 / preoperative body weight

^b^Extended dissection of nerve plexus around SMA was defined as more than a half

*RDI* Relative dose intensity, *N.R.* not reached

Boldface indicates the statistical significance

In the total cohort of 54 patients with surgically resected pancreatic cancer, BWL ≥10% tended to be related with worse DFS (26 months versus not reached; *p* = 0.218) and OS (46 months versus not reached; *p* = 0.163), respectively.

### Clinicopathological factors affecting adjuvant chemotherapy continuity

To determine factors that influence adjuvant chemotherapy continuity, 39 patients who received adjuvant chemotherapy were stratified according to RDI. Detailed clinicopathological characteristics of patients receiving adjuvant chemotherapy are presented in Table 4. Variables associated with RDI < 80% were age ≥ 75 years and BWL ≥10%. The incidence of postoperative diarrhea, clinically-relevant pancreatic fistula, and delayed gastric emptying were similar between the 2 groups. Multiple logistic regression analysis revealed BWL ≥10% as an independent predictable factor of adjuvant chemotherapy continuity (Table 5). Importantly, the severity of adverse events during adjuvant chemotherapy was similar between patients with BWL ≥10% and BWL < 10%. However, adjuvant chemotherapy discontinuation rate was significantly higher in patients with BWL ≥10% (50% vs 9.5%) (Table 6).

**Table 4:** Clinicopathological characteristics of patients with adjuvant chemotherapy

	RDI ≥ 80%	RDI < 80%	*p*
	*N* = 11	*N* = 28	
Age (mean ± SD)	63.4 ± 8.0	72.5 ± 6.1	< **0.001**
Age ≥ 75 (Y: N)	0: 11	11: 17	**0.017**
Sex (Male: Female)	5: 6	12: 16	N.S.
Comorbidity (Y: N)	6: 5	21: 7	N.S.
Operative method (PD: DP)	7: 4	21: 7	N.S.
Extended dissection of nerve plexus around SMA (Y: N)^a^	2: 9	10: 18	N.S.
Postoperative diarrhea (Y: N)	3: 8	10: 18	N.S.
Postoperative pancreatic fistula grade B or C	3: 8	8: 20	N.S.
Postoperative delayed gastric emptying (Y: N)	3: 8	4: 24	N.S.
Regimen (GEM: S-1: Both)	3: 6: 2	5: 20: 3	N.S.
Postoperative blood test			
WBC (/μl)	5238 ± 1588	5784 ± 1376	N.S.
Neutrophil	3528 ± 1333	3860 ± 1135	N.S.
Hg (g/dl)	11.2 ± 1.4	10.7 ± 1.1	N.S.
PLT (*10^4^/μl)	35.5 ± 11.7	32.5 ± 12.5	N.S.
AST (IU/l)	43.2 ± 23.0	35.1 ± 19.4	N.S.
ALT (IU/l)	65.9 ± 57.8	39.1 ± 30.0	N.S.
BUN (mg/dl)	12.6 ± 6.1	11.1 ± 3.1	N.S.
Cre (mg/dl)	0.65 ± 0.24	0.67 ± 0.20	N.S.
CCr (ml/min)	75.2 ± 25.1	62.2 ± 17.5	N.S.
Albumin (g/dl)	3.4 ± 0.3	3.1 ± 0.4	N.S.
Albumin maintenance rate ≥ 90% (Y: N)^b^	3: 8	6: 22	N.S.
BWL ≥10% (Y: N)^c^	2: 9	16: 12	**0.037**

^a^Extended dissection of nerve plexus around SMA was defined as more than a half

^b^Albumin maintenance rate was defined as: % albumin maintenance = albumin value at the time of discharge / preoperative albumin value × 100

^c^BWL (body weight loss): % body weight loss = (preoperative body weight – body weight at the time of discharge) × 100 / preoperative body weight

Boldface indicates the statistical significance

**Table 5:** Multiple logistic regression analysis for continuity of adjuvant chemotherapy

Variable	No.	OR (95% CI)	*p*-Value
Age			
< 75	28		
≥ 75	11		N.S.
BWL (%)^a^			
< 10	21		
≥ 10%	18	0.167 (0.030–0.910)	**0.039**

^a^BWL (body weight loss): % body weight loss = (preoperative body weight – body weight at the time of discharge) × 100 / preoperative body weight

Boldface indicates the statistical significance

**Table 6:** Adverse events during adjuvant chemotherapy

BWL < 10%	BWL ≥ 10%	*p*
*N* = 21	*N* = 18	
(Number: CTCAE Grade 4, 3, 2, 1)		
Anemia		
(0, 0, 1, 0)	(0, 0, 2, 0)	
Leucopenia		
(0, 1, 2, 0)	(0, 1, 3, 0)	
Neutropenia		
(0, 3, 2, 1)	(1, 1, 1, 0)	
Thrombopenia		
(0, 0, 1, 0)	(0, 0, 0, 0)	
Hepatic toxicity		
(0, 1, 3, 2)	(0, 0, 0, 0)	
Cholangitis		
(0, 0, 4, 0)	(0, 1, 1, 0)	
Diarrhea		
(0, 1, 1, 2)	(0, 1, 3, 2)	
Nausea		
(0, 0, 1, 0)	(0, 0, 3, 1)	
Eczema		
(0, 0, 1, 0)	(0, 0, 1, 1)	
Heart failure		
(0, 0, 1, 1)	(0, 1, 0, 0)	
Interstitial pneumonia		
(0, 0, 0, 1)	(0, 0, 0, 0)	
Others		
(0, 0, 0, 1)	(0, 0, 2, 0)	
Discontinuation of adjuvant chemotherapy (Y: N)		
2: 19	9: 9	**0.011**

*CTCAE* Common Terminology Criteria for Adverse Events

Boldface indicates the statistical significance

## Discussion

Pancreatic cancer is one of most challenging malignancies despite developments in surgery, chemotherapy and radiation therapy. Previously, numerous clinicopathological factors such as lymph node metastasis, portal vein invasion, surgical margin, and postoperative CA19–9 level have been reported as prognostic indicators in patients with resected pancreatic cancer [17–20]. In this retrospective analysis, we identified factors including extended dissection of the nerve plexus around the SMA, lymph node metastasis, residual tumor, and adjuvant chemotherapy of RDI < 80% as prognostic indicator. Additionally, we identified BWL after surgery as an influential factor of the continuity of adjuvant chemotherapy.

The CONKO-001 randomized trial compared postoperative prognosis between pancreatic cancer patients receiving adjuvant gemcitabine and those receiving surgery alone [10, 11]; the above study confirmed the benefit of gemcitabine-based adjuvant chemotherapy. Recently, the JASPAC-01 study showed that S-1 was superior to gemcitabine for both recurrence-free and overall survival [12]. More recently, the ESPAC-4 phase III trial showed the benefit of adjuvant chemotherapy using gemcitabine combined with capecitabine. While adjuvant chemotherapy has been widely accepted to improve patient prognosis, the dose intensity must be reduced or the chemotherapy must be discontinued because of various side effects. Therefore, it is important to identify risk factors that affect adjuvant chemotherapy continuity to improve the patient survival.

In gemcitabine-based adjuvant treatment, an inflammation-based prognostic score, the Glasgow Prognostic Score (GPS) was reported to be useful in predicting outcomes prior to adjuvant chemotherapy [21]. Aoyama et al. reported that CCr < 60 mL/min was a significant risk factor for the discontinuation of S-1 adjuvant chemotherapy, even if renal function was normal according to serum creatinine levels [22]. In our study, we identified that a RDI ≥80% was significantly associated with a favorable prognosis. Additionally, we found that postoperative BWL ≥10% was associated with a reduced RDI (< 80%).

BWL correlates with lower quality of life and is the most reliable indicator of malnutrition. An association between BWL and poor prognosis has been reported in various cancers. Previously, Fearon et al. reported that unresectable pancreatic cancer patients with a BWL ≥10% had a lower objective function and worse prognosis [23]. Recently, Hashimoto et al. reported that severe postoperative BWL following pancreatectomy was significantly associated with poor prognosis [24]. Furthermore, a decrease in lean body mass could also affect the incidence of chemotherapy-induced toxicities. For gastric cancer patients who underwent curative resection, BWL ≥15% was the most important risk factor for S-1 adjuvant chemotherapy compliance [25]. In our study, BWL ≥10% tended to be related with poor prognosis and significantly influenced adjuvant chemotherapy continuity. Our study also emphasizes the importance of adequate perioperative nutrition in patients who receive adjuvant chemotherapy.

Recently, Sho et al. reported that elderly patients aged ≥80 years were unlikely to complete the planned number of adjuvant chemotherapy cycles [26]. In our study, despite multiple logistic regression analysis not showing statistical significance, all patients ≥75 years had discontinued or reduced (RDI < 80%) adjuvant chemotherapy. Age is also a factor affecting adjuvant chemotherapy continuity for pancreatic cancer.

In this study, extended dissection of the nerve plexus around the SMA also had a negative effect on patient survival. Some randomized control trials have shown that extended lymphadenectomy, including dissection of the nerve plexus, has a minimal effect on patient survival. Farnell et al. reported that whole circumferential dissection of the nerve plexus around the SMA caused intractable diarrhea [27]. Recently, the International Study Group on Pancreatic Surgery (ISGPS) published a consensus statement regarding standard lymphadenectomy in surgery for pancreatic cancer. Complete resection around the SMA is not recommended [28]. BWL associated with diarrhea can cause nutritional and immunologic problems. Additionally, BWL may delay the initiation of postoperative chemotherapy.

This study has several potential limitations. The most significant one is that the results were derived from a retrospective single-center cohort with a small sample size. Next, the doses and intervals of the chemotherapy regimens varied among patients. With this taken into consideration, the results of this study should be verified in other large-scale series.

## Conclusions

Our results indicate that patient with adjuvant chemotherapy of RDI < 80% significantly influence patient prognosis. BWL ≥10% after surgery is predictive of chemotherapy discontinuation. Adequate perioperative nutritional and surgical interventions are necessary for patients who receive adjuvant chemotherapy for advanced pancreatic cancer. However, a further prospective validation study is needed to confirm these findings, as the present study was retrospective and utilized a small sample size.

## Abbreviations

BSA: Body surface area
BWL: Body weight loss
CCr: Creatinine clearance
DFS: Disease-free survival
ESPAC: European Study Group for Pancreatic Cancer
GPS: Glasgow Prognostic Score
ISGPS: International Study Group on Pancreatic Surgery
JASPAC: Japan Adjuvant Study Group of Pancreatic Cancer
OS: Overall survival
RDI: Relative dose intensity
S-1: Tegafur/gimeracil/oteracil
SMA: Superior mesenteric artery
SMV: Superior mesenteric vein
